# Structural characterization analysis and macromolecular model construction of coal from Qinggangping coal mine

**DOI:** 10.1038/s41598-023-40753-x

**Published:** 2023-09-01

**Authors:** Qi Li, Yujin Qin, Shaokui Ren

**Affiliations:** 1 College of Environment and Civil Engineering, Chengdu University of Technology, Chengdu, 610059 China; 2 Wenzhou University of Technology, Wenzhou, 325006 Zhejiang China; 3 State Key Laboratory of Coal Mine Safety Technology, China Coal Technology and Engineering Group Shenyang Research Institute, Shenfu Demonstration Zone, Shenyang, 113122 China

**Keywords:** Energy infrastructure, Mineralogy

## Abstract

Understanding the molecular structure characteristics of coal at the molecular level is of great significance to realize the rational utilization and efficient conversion of coal. This paper gives insights into the acquisition of characterization parameters of coal molecular microstructure by testing and analyzing the long flame coal from Qinggangping (QGP) Coal Mine through proximate analysis, ultimate analysis, vitrinite reflectance determination, fourier transform infrared Spectroscopy test (FTIR), X-ray photoelectron epectroscopy test (XPS), carbon nuclear magnetic resonance (^13^C-NMR) and X-ray diffraction (XRD). The results show that benzene rings in the QGP coal are mainly connected in a disubstituted way, accounting for 36.48%. Oxygen atoms mainly exist in the oxygen-containing functional groups such as the ether C–O, C=O and –COO. Aliphatic hydrocarbons in the aliphatic group are mainly of symmetrical -CH_x_ stretching vibration. Hydroxyl groups are mainly composed of OH–OH and OH–O hydrogen bonds, accounting for 29.21% and 21.53%, respectively. Nitrogen atoms exist in the form of C_4_H_5_N. The coal molecular is mainly of aromatic carbon structure, where the ratio of bridge aromatic carbon to peripheral carbon is 0.198. There are benzene, naphthalene and anthracene in the coal molecular structure, and the former two chemicals play a dominating role. According to the analysis results, the molecular formula of the QGP coal is finally determined as C_205_H_181_O_29_N_3_S. On this basis, the two-dimensional and three-dimensional macromolecular models are constructed with the assistance of simulation software. In addition, the ^13^C-NMR spectra and densities of the constructed molecular models are calculated, which verifies the rationality of the models. The macromolecular structure model of bituminous coal constructed in this study provides a theoretical model basis for the optimal surfactant.

## Introduction

Coal is a natural mixture, and its structure is complex, diverse and heterogeneous, and different coal ranks have different structures. The molecular structure of coal refers to the order and way in which different atoms in the molecule are connected to each other, and its structure determines the physical and chemical properties of coal and the substantial changes in the coal processing process. At the molecular level, it is formed by the condensation and connection of aromatic structures that contain aliphatic chains and various functional groups^1^. For a long time, scholars have adopted physical and chemical characterization methods to study the macromolecular structure of coal, and have put forward many coal structure models, such as the Fuchs Model^2^, the Given Model^3^, the Wiser Model^4^ and the Shinn Model^5^. Xiang et al.^6,7^ constructed the macromolecular structure models of coal from.

Yanzhou Coal Mine and anthracite from Chengzhuang Coal Mine by means of proximate/ultimate analysis and nuclear magnetic resonance (^13^C-NMR) spectroscopy and established practical molecular structure models by means of energy minimization simulation. Zhu et al.^8^ constructed the molecular structure model of lignite through molecular dynamics simulation software based on the experimental methods of ultimate analysis, nuclear magnetic resonance (^13^C-NMR) spectroscopy and X-Ray diffraction (XRD) spectroscopy. Tang et al.^9^ prepared coal-based graphene oxide through improved Hummers redox, and tested the prepared graphene oxide by ^13^C-NMR, scanning electron microscopy (SEM), energy dispersive spectroscopy (EDS) and X-ray photoelectron spectroscopy (XPS). Meanwhile, they constructed the molecular structure model of Jingxi coal-based graphene oxide.

The construction of coal macromolecular models becomes increasingly accurate and reliable as the instruments and equipment are continuously updated, which provides a reference for grasping the pyrolysis characteristics of coal^10^. To clarify the similarities and differences of macromolecular structures of different coals, advanced analysis technologies such as the proximate/ultimate analysis, Fourier transform infrared spectroscopy FTIR, XPS, XRD and ^13^C-NMR are adopted to characterize the organic matter of the coal from QGP Coal Mine (hereafter referred to as the QGP coal) and obtain information on their functional group composition, carbon skeleton structures and surface element composition^11^. The macromolecular structure model of coal was constructed, and the ^13^C-NMR and density simulation calculation of the molecular model was carried out to verify the rationality of the built model.

The needle radius is 0.13 nm (the molecular dynamics radius of He), the unit cell model of the coal macromolecular structure and the pore size distribution are obtained. It lays a foundation for studying the adsorption performance of coal molecules from the perspective of molecular dynamics. Moreover, it also provides convenience for researchers to consult and refer to the construction approaches and methods of the macromolecular structure models of coal samples.

## Coal sample preparation and experimental methods

### Coal sample preparation

The coal sample was prepared in strict accordance with the provisions of *Method for Preparation of Coal Sample* (GB474-1996). QGP coal on the air-dried basis was used as the raw material which was repeatedly crushed and sieved by a crusher and a screen mesh for preparing the coal sample with a particle size of more than 200 meshes. Then, 200 g of sample were randomly selected for testing^12^.

### Industrial analysis and elemental analysis testing

The industrial analysis is determined according to the national standard Industrial Analysis Method for Coal (GB/T 212-2008), and the elemental analysis of coal samples is based on the national standard Determination of carbon and hydrogen in coal (GB/T 476-2008), Coal Methods for the Determination of Nitrogen in Coal (GB/T 19227-2008) and Method for the Determination of Total Sulfur in Coal (GBJT 214-2007) were determined by Vario EL element analyzer from EA Company in Germany.

### Fourier transform infrared spectroscopy test (FTIR)

The Thermo Scientific Nicolet iS5 FTIR spectrometer was used, which recorded a spectral range of 4000–400 cm^−1^, a moving mirror speed of 0.4747, and a resolution of 0.04 cm^−1^. Add 0.2 g of potassium bromide to the test sample for mixing and make transparent thin slices with a thickness of 0.2–0.5 mm. The number of scans is 16 to obtain the infrared spectrum of the coal sample.

### X-ray photoelectron epectroscopy test (XPS)

In the test, ESCALAB250 X-ray photoelectron spectrometer was used to test the element types and chemical valence states on the surface of coal samples, and the full spectrum was scanned, and the C, O, and N elements were narrowly scanned. The parameters are set as follows: the initial pressure is 10^–4^ kPa, the full scan transmission energy is 150 eV, the step size is 0.5 eV; the AlKα anode power is 200 W; the narrow scan transmission energy is 60 eV, the step size is 0.05 eV.

### Nuclear magnetic resonance spectroscopy test (^13^C-NMR)

The Varian INOVA300 superconducting NMR instrument was used for the test, and the parameters were set as follows: the magic angle speed was 8000 kHz, the sampling time was 0.05 s, the cycle time was 5 s, the pulse width was 4.2 μs, the scanning was 3000–5000 times, and the resonance of the carbon signal was detected. The frequency is 75.43 MHz. The cross-polarization technique was used with a contact time of 5 ms and the spectral width of 30,000 Hz.

### X-ray diffraction (XRD)

The XRD experiment was carried out by the D8 ADVANCE X-ray diffractometer manufactured by Bruker, Germany. Experimental conditions are tube pressure 40 kV, tube flow 200 μA, Cu target, diffraction width DS = SS = 1°, RS = 0.3 mm, scanning speed 2000 (d min^−1^), scanning range 10°–80°.

## Results and analysis

### Proximate/ultimate analysis

The contents of C, H, O, N and S elements of the QGP coal sample are shown in Table 1. As its vitrinite reflectance R°max is 0.52%, the QGP coal sample selected in this paper belongs to long flame coal^13^.

**Table 1:** Industrial analysis and elemental analysis of coal samples in Qing gang ping coal mine.

*R°*_*max*_/%	Proximate analysis/% (mass)			Ultimate analysis/% (mass, daf)				
	M_ad_	A_d_	V_daf_	C	H	O	N	S
0.52	2.43	13.06	28.43	77.75	5.07	14.54	1.43	1.21

### Analysis on FTIR results

**Figure 1:**
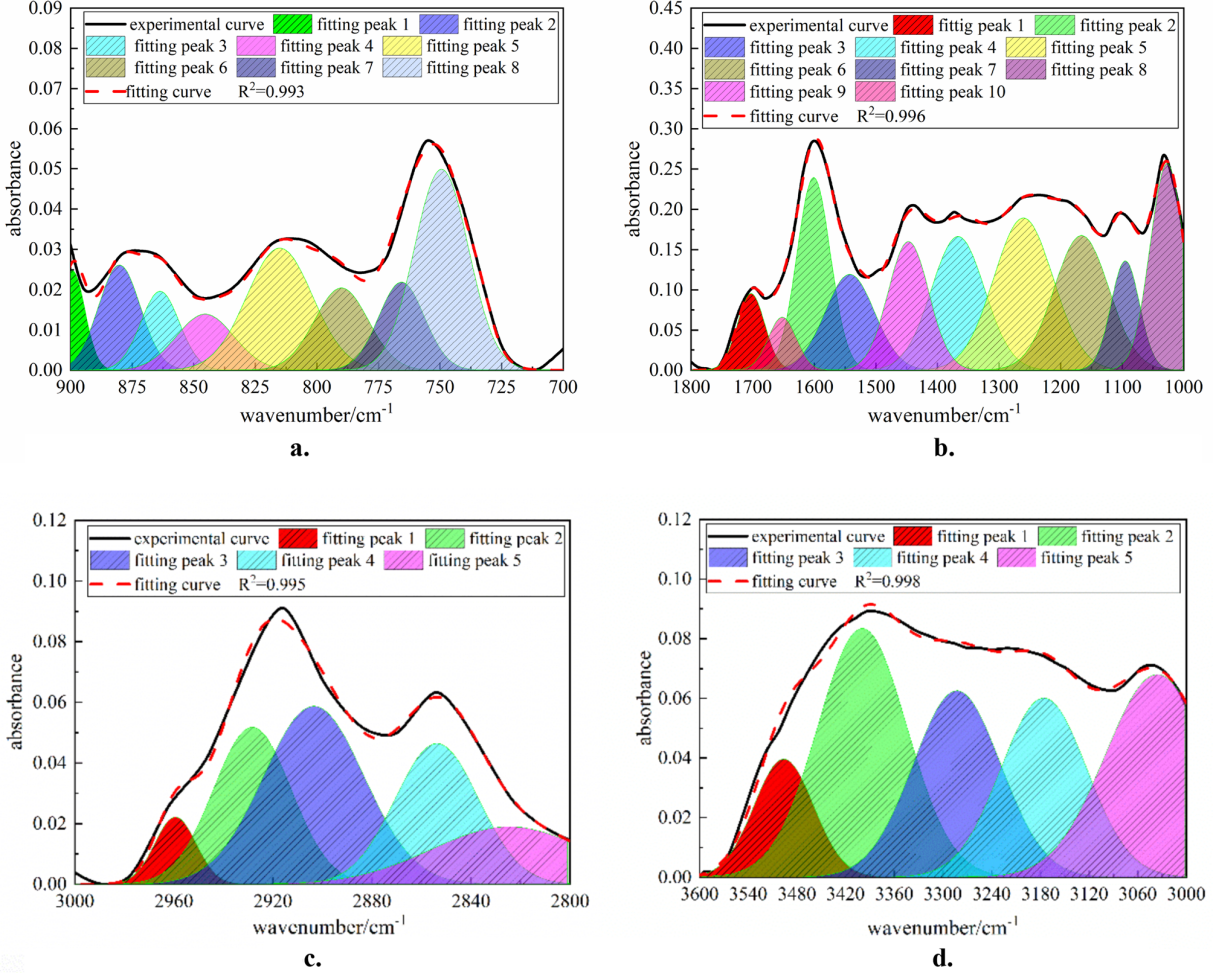
FTIR spectral peak fitting of the QGP coal. (**a**) FTIR spectral peak fitting (900–700 cm^−1^). (**b**) FTIR spectral peak fitting (1800–1000 cm^−1^). (**c**) FTIR spectral peak fitting (3000–2800 cm^−1^). (**d**) FTIR spectral peak fitting (3600–3000 cm^−1^).

The positions and intensities of absorption peaks in the FTIR spectrum, which are subject to the vibration form of each group in the molecule and the influences from adjacent groups, can reflect the structural characteristics of the molecule. The fitting of the absorption peak intensity can be used to quantitatively analyze the molecular composition or the content of functional groups^14^. The FTIR spectrum can be divided into four absorption bands. Among them, the 3600–3000 cm^−1^ band is the hydroxyl group absorption band; the 3000–2800 cm^−1^ band is attributed to the aliphatic hydrocarbon absorption; the 1800–1000 cm^−1^ band belongs to the oxygen-containing functional group; and the 900–700 cm^−1^ band is the aromatic hydrocarbon absorption band^15^. The peak analysis module in Origin software is used to fit and analyze the FTIR test results in Fig. 1.

On the basis of peak fitting, the parameters of infrared absorption peaks in each band are calculated and analyzed in Table 2.

**Table 2:** FTIR absorption peak parameters of the QGP coal.

No	Peak position/cm^−1^	Peak area	Relative area/%	Attribution
(a) 900–700 cm^−1^				
1	896.21	0.291	5.55	5-substituted benzene ring (1H)
2	880.16	0.556	10.61	5-substituted benzene ring (1H)
3	863.65	0.386	7.37	5-substituted benzene ring (1H)
4	845.38	0.411	7.85	5-substituted benzene ring (1H)
5	815.07	1.121	21.39	3 or 4-substituted benzene ring (3H/2H)
6	790.04	0.563	10.75	3 or 4-substituted benzene ring (3H/2H)
7	765.61	0.512	9.77	2-substituted benzene ring (4H)
8	749.42	1.399	26.70	2-substituted benzene ring (4H)
(b) 1800–1000 cm^−1^				
1	1702.55	5.828	4.38	Stretching vibration of –COO in carboxylic acid
2	1651.88	3.392	2.55	Stretching vibration of C=O induced by the conjugative effect
3	1600.91	16.262	12.22	Skeleton vibration of C=C in aromatic hydrocarbon
4	1542.27	12.067	9.07	Skeleton vibration of C=C in aromatic hydrocarbon
5	1447.01	13.323	10.01	Asymmetric deformation vibration of CH_3_– and CH_2_–
6	1366.49	18.033	13.55	Symmetric bending vibration of CH_3_–
7	1260.38	22.904	17.21	Stretching vibration of C–O in phenol, alcohol, ether, phenoxy and ester
8	1166.07	18.71	14.07	Stretching vibration of C–O in phenol, alcohol, ether, phenoxy and ester
9	1094.91	7.907	5.94	Stretching vibration of C–O in phenol, alcohol, ether, phenoxy and ester
10	1027.14	14.771	11.1	Ash content
(c) 3000–2800 cm^−1^				
1	2959.39	0.635	7.37	Asymmetric stretching vibration of CH_3_
2	2928.35	2.030	22.56	Asymmetric stretching vibration of CH_2_
3	2903.50	2.820	32.73	Stretching vibration of CH
4	2853.79	2.047	23.76	Symmetric stretching vibration of CH_3_
5	2834.64	1.169	13.57	Symmetric stretching vibration of CH_2_
(d) 3600–3000 cm^−1^				
1	3506.25	3.632	9.24	OH–π hydrogen bond
2	3399.57	11.477	29.21	OH–OH hydrogen bond
3	3282.88	8.458	21.53	OH–O hydrogen bond
4	3175.77	7.964	20.27	Cyclic hydrogen bond
5	3035.91	7.756	19.74	OH–N hydrogen bond

For Fig. 1a absorption peaks of the aromatic structure (900–700 cm^−1^)^16^, according to Table 2a, the peak areas of the 5-substituted benzene ring, the 3-substituted or 4-substituted benzene ring, and the disubstituted benzene ring in the QGP coal sample are 1.644, 1.684 and 1.911, respectively, and the corresponding relative area ratios are 31.38%, 32.14% and 36.48% respectively. Such a result shows that most benzene rings in the macromolecular structure of the QGP coal sample are disubstituted functional groups, and few are 3-substituted, 4-substituted and 5-substituted. For Fig. 1b absorption peaks of the oxygen-containing functional group (1800–1000 cm^−1^)^17^, according to Table 2b, the peak areas of the C–O stretching vibration in the phenol, alcohol, ether, phenoxyl and ester, aromatic (C=C) and carbonyl group in the QGP coal sample are 49.52, 28.329 and 9.22, respectively, and the corresponding relative area ratios are 37.22%, 21.29% and 6.93%, respectively. For Fig. 1c absorption peaks of the hydroxyl structure (3000–2800 cm^−1^) 18, according to Table 2c, most aliphatic hydrocarbons in the QGP coal sample are of symmetrical –CH_x_ stretching vibration, and few are of –CH stretching vibration, wherein the former has a peak area of 3.216 and a relative area ratio of 37.33% and the latter has a peak area of 2.820 and a relative area ratio of 32.73%. The peak of symmetrical CH_3_ is higher than that of symmetrical CH_2_ and the peak of antisymmetric CH_2_ is higher than that of antisymmetric CH_3_, which shows that the aliphatic chains in the molecular structure of the sample are mainly long chains. For Fig. 1d absorption peaks of the hydroxyl group (3600–3000 cm^−1^)^19^, according to Table 2d, the total hydroxyl groups of the QGP coal sample are mainly composed of OH–OH hydrogen bonds with a peak area of 11.477 and a relative area ratio of 29.21%, followed by OH–O hydrogen bonds, cyclic hydrogen bonds, OH–N hydrogen bonds and OH–π hydrogen bonds whose peak areas are 8.458, 7.964, 7.756 and 3.632 and relative area ratios are 21.53%, 20.27%, 19.74% and 9.24%, respectively.

### Analysis on XPS results

**Figure 2:**
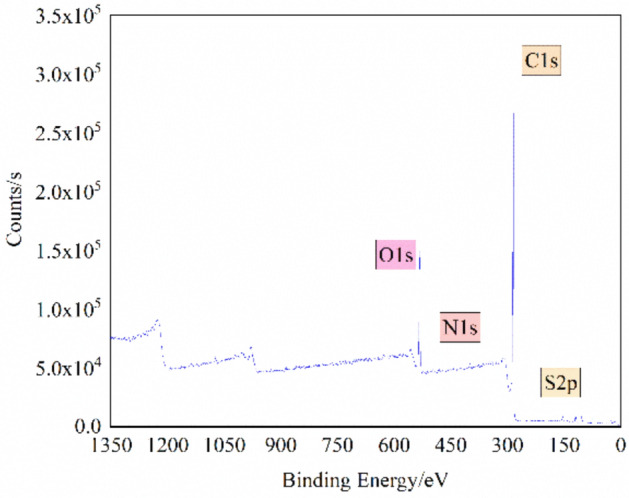
XPS structure of the QGP coal.

Nitrogen and sulfur exist in the macromolecular structure of coal in the form of functional groups^20^. XPS full-spectrum scanning was adopted for the QGP coal sample, and the results are shown in Fig. 2. Furthermore, Origin software was used for peak fitting to analyze the different states of nitrogen and sulfur, so as to obtain more accurate information about the content of surface functional groups of nitrogen and sulfur.

### Analysis on nitrogen in the QGP coal sample

When XPS is adopted for the analysis of the structures of various forms of nitrogen in coal, it is generally considered that four characteristic peaks, i.e., C_5_H_5_N, C_4_H_5_N, –N–(CH_3_)_3_ and N_x_O_y_, can be fitted^21^. The peak binding energy values of the four peaks are (398.8 ± 0.4) eV, (400.2 ± 0.3) eV, (401.4 ± 0.3) eV and (402.9 ± 0.5) eV, respectively.

**Figure 3:**
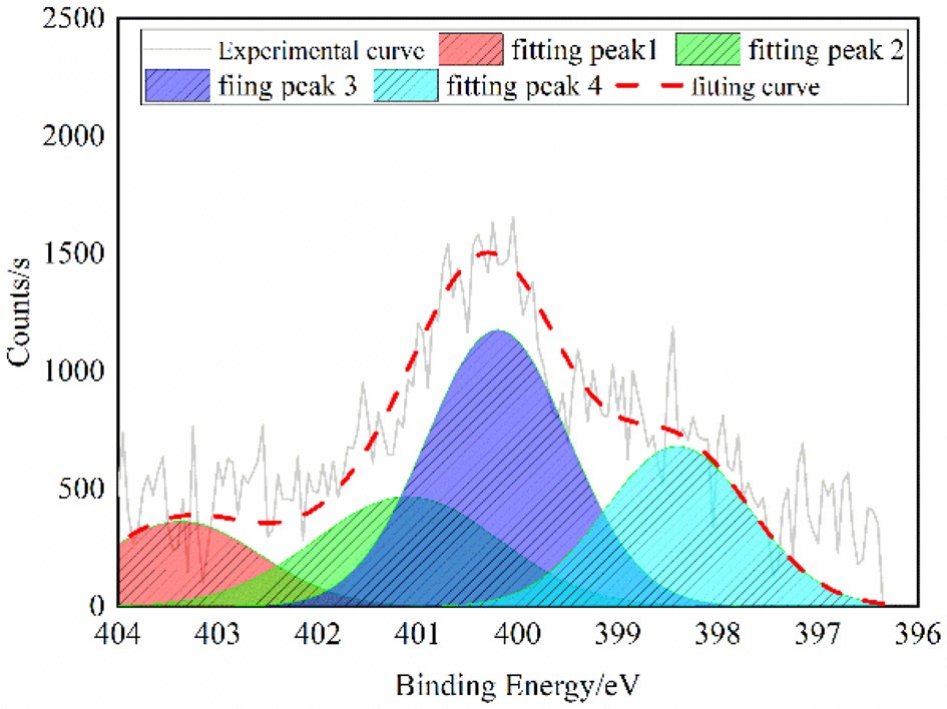
XPS-N1s spectrum of the QGP coal.

As the measured XPS data fluctuate greatly, they need to be smoothed prior to peak fitting, and the XPS-N1s peak fitting results of the QGP coal sample are illustrated in Fig. 3.

The results in Table 3 show that C_5_H_5_N and C_4_H_5_N are the primary existing forms of nitrogen in the QGP coal sample, playing a dominant role, while the contents of -N-(CH_3_)_3_ and N_x_O_y_ are relatively low.

**Table 3:** XPS-N1s spectral data of the QGP coal.

Attribution	Pyrrole nitrogen	Pyridine nitrogen	Quaternary nitrogen	Nitrogen oxide
Area Fit TP ωmol/%	24.76	42.28	21.57	11.39

### Analysis on sulfur in the QGP coal sample

When XPS is adopted for the analysis of the structures of various forms of sulfur in coal, it is generally believed that there are four kinds of sulfur^22^. They are the mercaptan and thioether type, the thiophene type, the sulfone and sulfoxide type and the inorganic sulfur type whose peak binding energy values are (162.2–164) eV, (164–164.4) eV, (165–168) eV and (169–171) eV, respectively.

**Figure 4:**
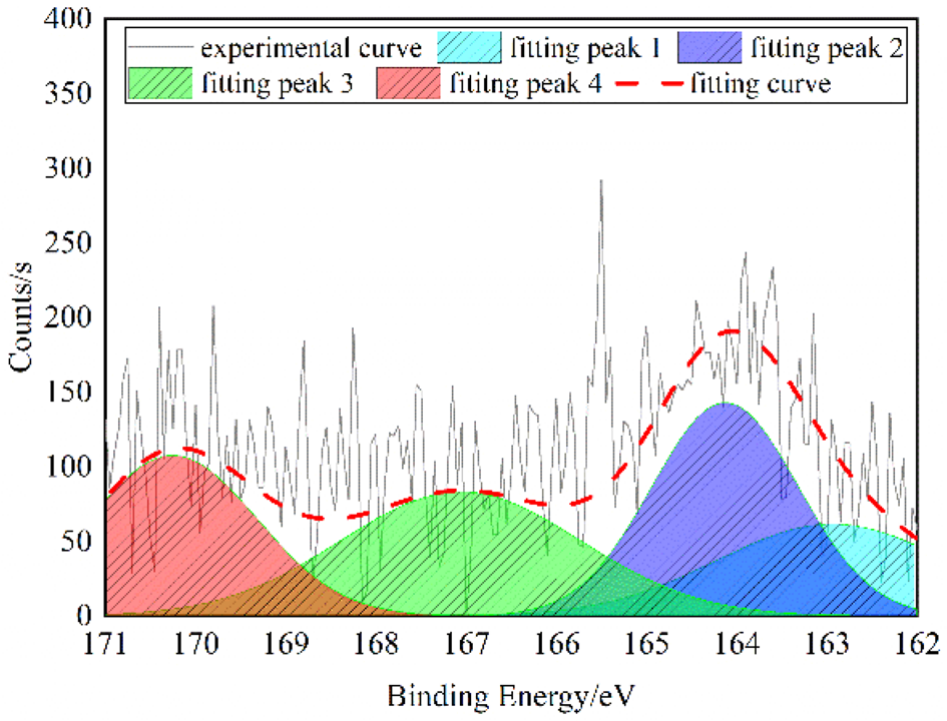
XPS-S2p spectrum of the QGP coal.

Also, the significantly fluctuating measured XPS data need to be smoothed before peak fitting, and the XPS-S2p peak fitting results of the QGP coal sample is shown in Fig. 4.

The results in Table 4 suggest that sulfur in the molecular structure of the QGP coal sample exists mostly in the form of thiophene, sulfone and sulfoxide and rarely in the form of inorganic sulfur.

**Table 4:** XPS-S2p spectral data of the QGP coal.

Attribution	Mercaptan thiophenol	Thiophene type sulfide	Sulfoxide or sulfone type sulfur	Inorganic sulfur
Area fit TP ωmol/%	16.56	32.58	30.21	20.65

### Analysis on ^13^C-NMR results

Carbon atoms constitute the main macromolecular skeleton of coal, and other groups are connected to carbon atoms skeleton in different ways. Different types of carbon atoms (aliphatic carbon, aromatic carbon, etc.) and different functional groups connected to them bring about varying chemical shifts of the corresponding ^13^C-NMR peaks^23^. ^13^C-NMR can quantitatively analyze the structural composition of organic materials, and its peak position determines its corresponding chemical shift. The resulting information on carbon skeleton of the coal sample can be used to study the macromolecular structural characteristics of coal vitrinite. According to previous research results^6,24,25^, the structural attribution of chemical shifts of carbon atoms is given in Table 5.

**Table 5:** Assignment of chemical shifts of carbon element.

Chemical shift/ppm	Attribution	Chemical shift/ppm	Attribution
16	R–CH_3_	75–90	R-O-R
20	Ar–CH_3_	100–129	Ar–H
23	CH_2_–CH_2_	129–137	Bridgehead
33	CH_2_	137–148	Ar–C
36–50	C, CH	148–165	Ar–O
50–60	O–CH_3_, O–CH_2_	165–190	COOH
60–70	O–CH	190–220	C=O

**Figure 5:**
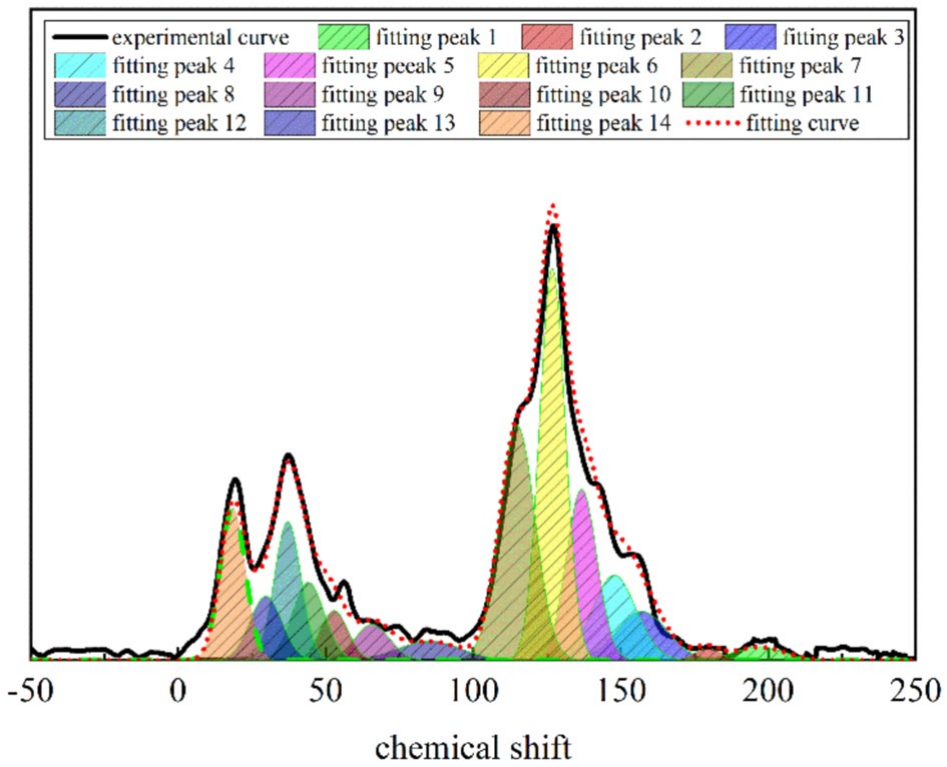
^13^C-NMR peak fitting spectrum of the QGP coal.

The chemical shifts of the spectrum (− 50 to 200 ppm) are peak fitted by Origin in Fig. 5, and the peaks are classified and analyzed according to the chemical shift structure of the carbon element in Table 6.

**Table 6:** ^13^C-NMR peak fitting spectrum.

No.	Chemical shift/ppm	Relative area/%	Attribution
1	197.02	0.76	Carbonyl carbon
2	179.40	1.23	Carbonyl carbon
3	157.14	6.92	Oxygen-substituted aromatic carbon
4	147.77	8.96	Side-branched aromatic carbon
5	136.70	12.19	Bridged aromatic carbon
6	126.80	24.69	Protonated aromatic carbon
7	114.93	20.88	Protonated aromatic carbon
8	85.17	1.57	Oxygen-grafted aliphatic carbon
9	65.76	1.68	Oxygen-grafted aliphatic carbon
10	52.82	1.94	Oxygen-grafted aliphatic carbon
11	44.11	3.86	Methylene and quaternary carbon
12	37.11	5.29	Methylene and quaternary carbon
13	29.73	3.39	Methylene and quaternary carbon
14	18.49	6.64	Aliphatic (aromatic) methyl

The fitting results show that ^13^C-NMR of the QGP coal sample is mainly composed of two peak groups, namely aliphatic carbon (*f*_al_ = *f*_al_^*^ + *f*_al_^H^ + *f*_al_^O^) (16–90 ppm) and aromatic carbon (*f*_a_^’^ = *f*_a_^H^ + *f*_a_^N^ = *f*_a_^H^ + *f*_a_^P^ + *f*_a_^S^ + *f*_a_^B^) (90–165 ppm). The oxygen-containing functional groups in the coal macromolecular structure are mainly oxygen-grafted aromatic carbon (*f*_al_^P^) and oxygen-grafted aliphatic carbon (*f*_al_^O^).

Through the summary of ^13^C-NMR test results, the list of carbon-containing types shown in Table 7 is listed.

**Table 7:** Structural parameters determined by ^13^C-NMR of the QGP coal.

Sample	*f*_al_*	*f*_*al*_^*H*^	*f*_*al*_^*O*^	*f*_*a*_^*H*^	*f*_*a*_^*B*^	*f*_*a*_^*S*^	*f*_*a*_^*P*^	*f*_*a*_^*N*^	*f*_*a*_^*C*^	*f*_*al*_	*f*_*a*_	*f*_*a*_*’*
QGP	6.64	12.54	5.19	45.57	12.19	8.96	6.92	28.07	1.99	24.37	75.63	73.64

*f*_al_* is methyl carbon/quaternary carbon; *f*_*al*_^*H*^ is methylene/methylene carbon; *f*_*al*_^*O*^ is oxygen-grafted aliphatic carbon; *f*_*a*_^*H*^ is protonated aromatic carbon; *f*_*a*_^*B*^ is bridge aromatic carbon; *f*_*a*_^*S*^ is side branched aromatic carbon; *f*_*a*_^*P*^ is oxygen-grafted aromatic carbon; *f*_*a*_^*N*^ is non protonated aromatic carbon; *f*_*a*_^*C*^ is carbonyl/carboxyl carbon; *f*_*al*_ is aliphatic carbon; *f*_*a*_ is total sp2 hybrid carbon; and *f*_*a*_^*’*^ is aromatic carbon^6^.

The ratio of bridge aromatic carbon to peripheral carbon (*X*_*BP*_) is used to characterize the polycondensation degree of the aromatic structure in coal macromolecular structure^26^ and the calculation formula is as follows:

1 $$ X_{BP} = \frac{{f_{a}^{B} }}{{f_{a}^{H} + f_{a}^{P} + f_{a}^{S} }} $$

By substituting relevant parameters into the formula, the calculated $X_{BP}$ equals 0.1983≈0.2. The ratio of bridge aromatic carbon to peripheral carbon can provide theoretical support for the construction of the coal macromolecular structure model.

### X-ray diffraction analysis

**Figure 6:**
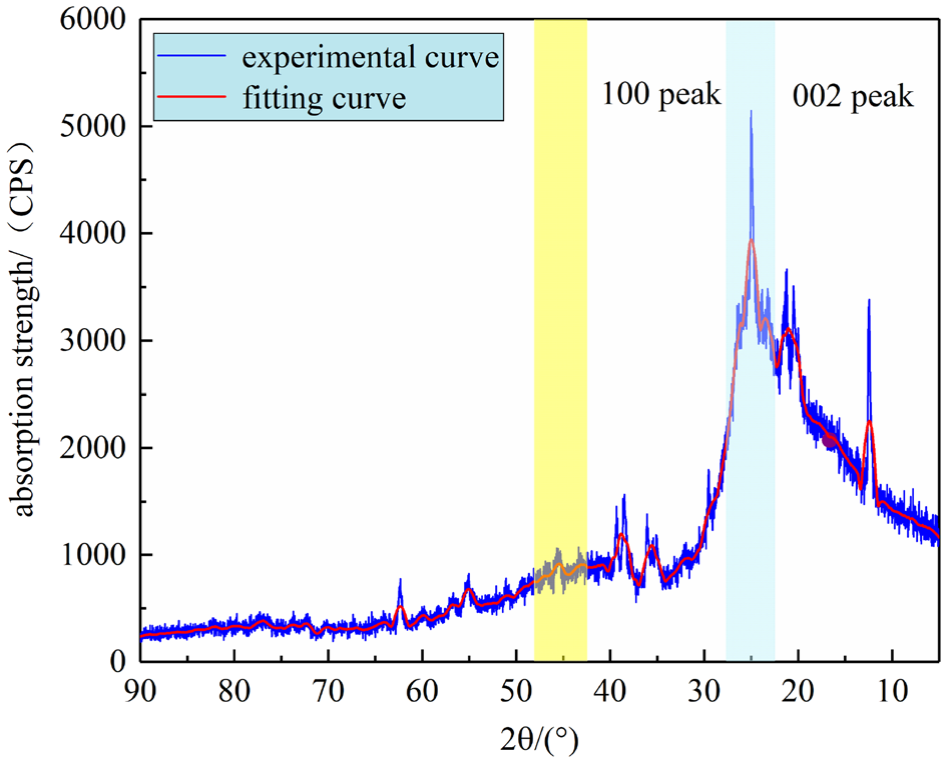
XRD spectrum of the QGP coal.

In this paper, changes of the microcrystalline structure of the QGP coal sample are analyzed based on the changes of structure parameters of the XRD spectrum. As presented in the XRD spectrum of the QGP coal sample in Fig. 6, there are two broad peaks when 2θ = 20° ~ 30° and 2θ = 40° ~ 50°, both of which belong to organic matter. When 2θ = 20° ~ 30°, the peak, corresponding to the 002 surface of the microcrystalline structure, is called the 002 peak. It is superimposed by the γ band and the 002 band^27^. The 002 band of the coal sample is related to the distance between aromatic rings, and the γ band is related to the aliphatic groups (including aliphatic branched chain and alicyclic ring) in the molecular structure. When 2θ = 40° ~ 50°, the peak, corresponding to the 100 peak of the microcrystalline structure, characterizes the condensation of aromatic rings in the coal sample, i.e., the size of the aromatic carbon mesh layer. In addition, some sharp high-intensity peaks can be observed in the XRD spectrum, which are caused by minerals such as calcite (near 2θ = 30°), kaolinite (2θ = 25°) and quartz (2θ = 21°) in the coal^28^.

**Figure 7:**
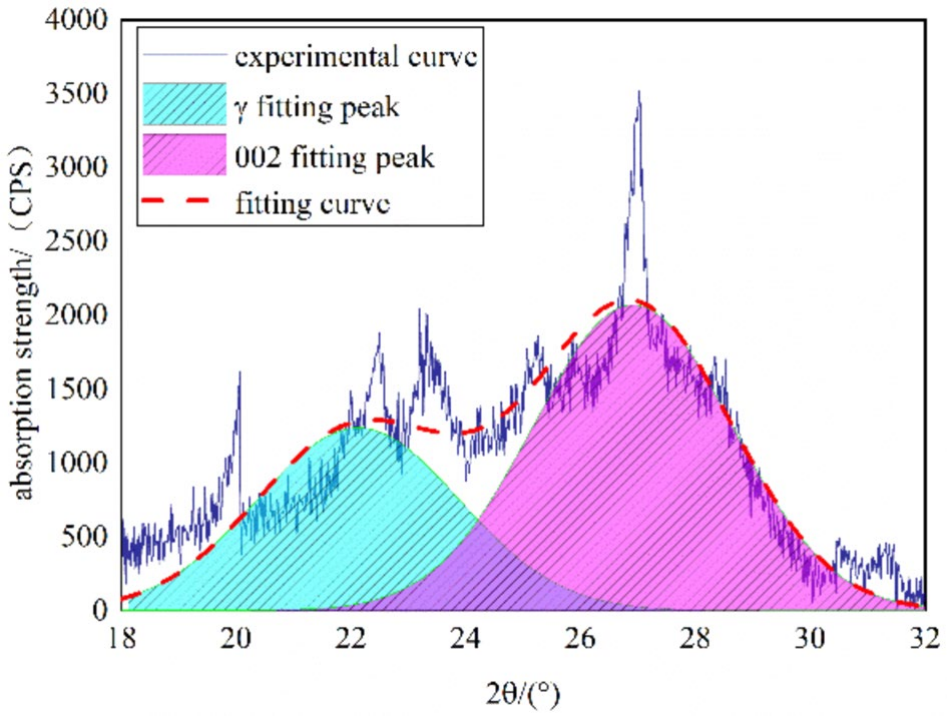
XRD-002 peak fitting spectrum of the QGP coal.

To further study the law of XRD microcrystalline structure parameters of the coal sample, Origin was adopted to smooth the original data firstly, and then the 002 diffraction peaks of the coal sample were peak fitted to obtain the regularly arranged 002 peaks for the analysis of microcrystalline structure parameters. The fitting results are shown in Fig. 7.

The fitting results show that the γ peak is narrow and low while the 002 peak is wide and high. Based on these parameters, the microcrystalline structure parameters of coal, including the layer spacing *d*, the ductility of the aromatic layer *La*, the stacking degree *Lc*, the number of the stacking aromatic layers *Nave* and the aromaticity *f*_*a*_, can be calculated through the Bragg equation and the Scherrer formula^29^. The calculation results are shown in Table 8.

2 $$ \left\{ \begin{gathered} d_{002} = \frac{\lambda }{{2\sin \theta_{002} }} \hfill \\ L_{a} = \frac{1.84\lambda }{{\beta_{100} \cos \theta_{100} }}L_{c} = \frac{0.94\lambda }{{\beta_{002} \cos \theta_{002} }} \hfill \\ f_{a} = \frac{{A_{002} }}{{A_{\gamma } + A_{002} }}N_{ave} = \frac{{L_{c} }}{{d_{002} }} \hfill \\ \end{gathered} \right. $$

where λ is the diffraction wavelength of X-ray, being 0.154 nm; θ002 and θ100 are the positions of the 002 diffraction peak and the 100 diffraction peak, respectively; β002 and β100 are the fitting half peak widths of the 002 peak and the 100 peak, respectively; Aγ and A002 are the areas of the γ peak band and the 002 peak band, respectively.

**Table 8:** Microcrystalline structure parameters of the QGP coal.

Sample	θ002/(°)	θ100/(°)	d002/nm	La/nm	Lc/nm	fa	Nave
QGP	12.59	21.59	0.3533	1.38	2.09	0.57	5.92

## Construction and optimization of the coal macromolecular structure models

### Construction of the two-dimensional macromolecular model

When the content of carbon lies in the range of 70–83%, the average number of condensation rings of an aromatic structural unit is 2^30^. The ratio of bridge aromatic carbon to peripheral carbon of the QGP coal sample is 0.20. This ratio reveals that most aromatic carbons exist in the form of benzene and naphthalene, and few exist in the form of anthracene. Through Matlab programming calculation, the type and number of aromatic structural units of the model whose ratio of bridge aromatic carbon to peripheral carbon is closest to the experimental ratio are obtained, and the aromatic skeleton combination in the structural model is determined (Table 9). In this case, the total number of aromatic carbons in the model is 151, accounting for 73.64% according to ^13^C-NMR. Hence, the total number of carbons in the molecular structure of the coal sample is 205, and that of aliphatic carbons and (carboxyl) carbonyl carbons in the molecular structure of coal is 54. The ultimate analysis of the coal sample shows that the contents of carbon, oxygen, nitrogen and sulfur of the coal sample are 77.75%, 14.54%, 1.43% and 1.21% respectively. Therefore, it can be calculated that the numbers of oxygen, nitrogen and sulfur in the macromolecular structure of the coal sample are 29, 3 and 1, respectively. The XPS experimental analysis results indicate that nitrogen exists in the QGP coal sample mainly in the form of C_5_H_5_N and C_4_H_5_N, and the ratio of the numbers of the two is 1: 2. Therefore, the numbers of C_5_H_5_N and C_4_H_5_N in the QGP coal sample are 1 and 2, respectively. Sulfur exists in the QGP coal sample mainly in the form of thiophene sulfur, whose content is 32.58%. In order to construct a typical model, a sulfur atom in the form of thiophene sulfur is constructed in the coal molecule. According to the FTIR analysis on the contents of oxygen-containing functional groups, the ratio of phenol, alcohol and ether (C–O) to carboxyl and carbonyl (C=O) in the oxygen-containing functional groups of the QGP coal sample is about 5.37:1, and the content of carboxyl is high. Therefore, the numbers of carboxyl and carbonyl in the QGP coal sample are 3 and 1, respectively. In addition, based on the ^13^C-NMR test, the content ratio of oxygen-substituted carbon to oxygen-grafted aliphatic carbon is about 1.3:1, so it can be determined that there are 13 hydroxyl (–OH) radicals and 9 oxygen-grafted aliphatic carbons in the QGP coal sample.

**Table 9:** Existing form of macromolecular configuration aromatic carbon in the QGP coal.

Attribution	Pyrrole nitrogen	Pyridine nitrogen	Thiophene type sulfide	Benzene	Naphthalene	Anthracene
Count	1	2	1	5	9	1

**Figure 8:**
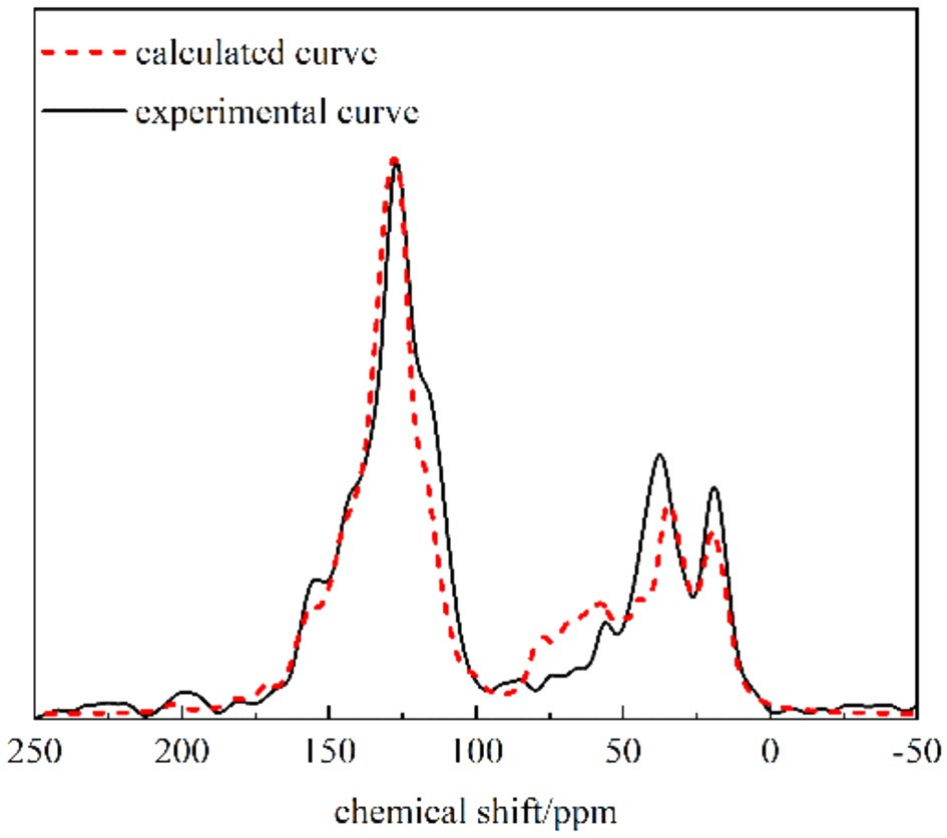
Experimental and calculated ^13^C-NMR spectra of the QGP coal.

**Figure 9:**
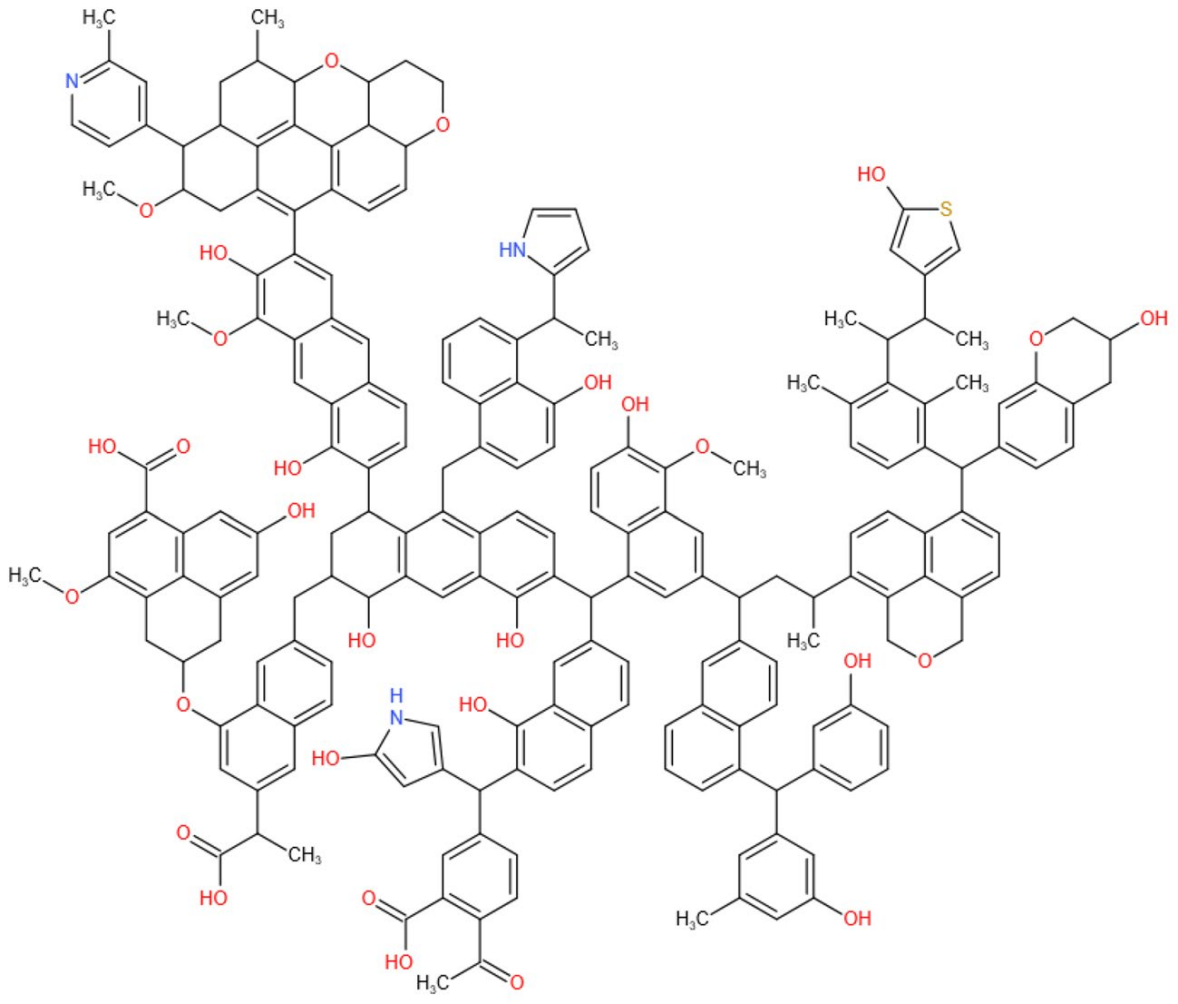
Macromolecular structure model of the QGP coal.

Based on the above analysis, the macromolecular model of the QGP coal sample was constructed with the aid of the chemical drawing software Kingraw and then imported into the MestReNova software. By constantly adjusting the positions and connection modes of various groups in the coal molecule, the aromatic unit and aromaticity were kept unchanged. Finally, the calculated ^13^C-NMR spectrum of the constructed model was compared with the experimental one in Fig. 8. The comparison results show that the ^13^C-NMR spectrum of the constructed macromolecular model of the QGP coal sample is consistent with the experimental one; that is, it can well reflect the macromolecular structure of the studied coal sample. In conclusion, the molecular formula of the QGP coal sample is C_205_H_181_O_29_N_3_S (C: 77.38, S: 1.01, N: 1.32, O: 14.60, H: 5.69), and its molecular model is shown in Fig. 9.

### Construction of the three-dimensional macromolecular model

**Figure 10:**
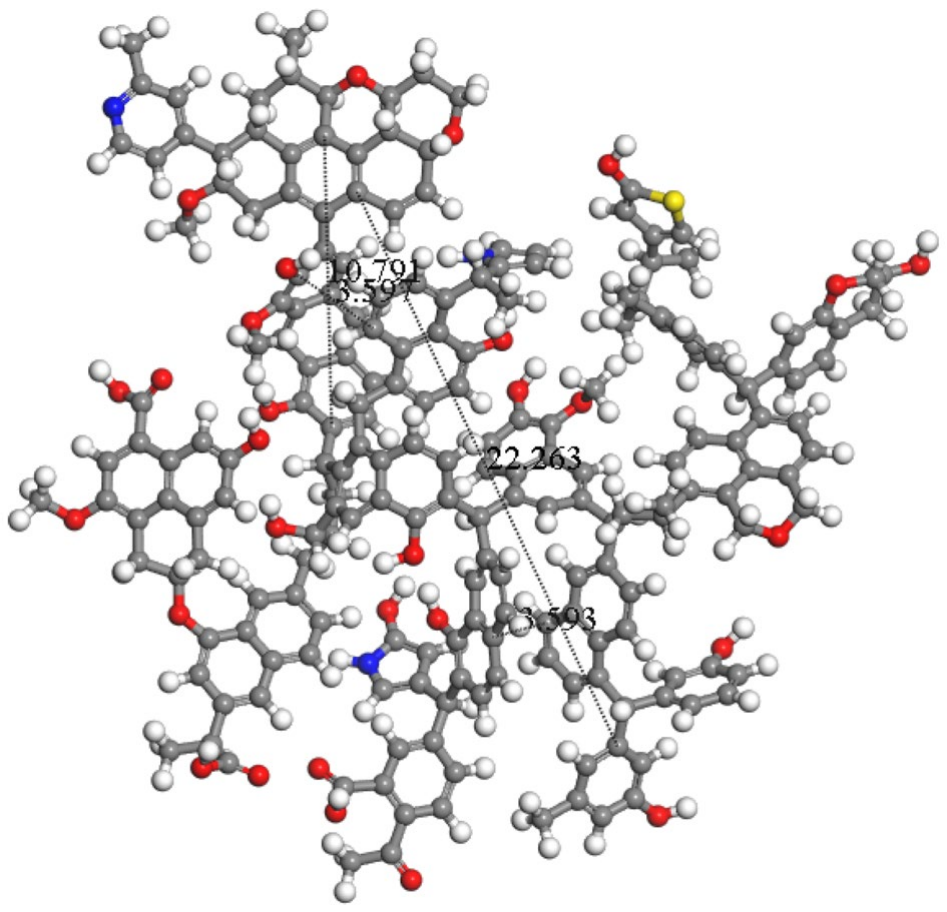
Geometric configuration of the QGP coal structure.

The two-dimensional planar model of coal molecule was imported into the molecular dynamics simulation software MaterialsStudio (hereinafter referred to as MS). Afterwards, H was added until it gets saturated. Following this, the GeometryOptimization module was used to optimize the structure of the macromolecular model of the coal sample, where the field force was COMPASS; the calculation accuracy was Fine; and the maximum iterative step was 2000. The obtained macromolecular structure model of the coal sample is shown in Fig. 10. By means of Measure, the measured d002 of the QGP coal in parallel arrangement is 0.3597 nm; Lc is 2.2263 nm; La is 1.0791 nm. These values are close to the data measured by XRD, i.e., d002 is 0.3533 nm; Lc is 2.09 nm; and La is 1.38 nm. This further shows that the macromolecular three-dimensional model of the coal sample is reasonable.

The structure of the constructed three-dimensional macromolecular model was optimized as follows. First, the AmorphousCell module was adopted; and the calculation accuracy and the force field were set as Medium and COMPASS, respectively. Subsequently, they were put into the crystal cell where a limit of three-dimensional periodic boundary conditions was added and the density was set as 1.17 g/cm^3^. Following this, the GeometryOptimization module was adopted to optimize the structure of the cell model of three types of coal samples, with the force field set as COMPASS, the calculation accuracy as Fine, and the maximum iterative step as 2000. Next, it underwent the dynamic processing of the Anneal module (the force field was COMPASS; the calculation accuracy was Fine; the temperature was 300–600 K; NPT dynamic ensemble; 5 cycles; the number of time steps was 1 fs; and the total simulation time was 1000 ps) and the Danymics module (the force field was COMPASS; the calculation accuracy was Fine; NPT dynamic ensemble, the number of time steps was 1 fs; and the total simulation time was 1000 ps)^31^. In this way, the energy of the coal in the structural model decreased to the minimum and finally tended to level off, and the model density stabilized at 1.13 g/cm^3^. This value is close to the experimental one, but it is smaller than the actual value. This phenomenon is caused by the absence of mineral elements (such as kaolinite, calcite and quartz) in the model. Therefore, the density of the model is considered to be reasonable.

**Figure 11:**
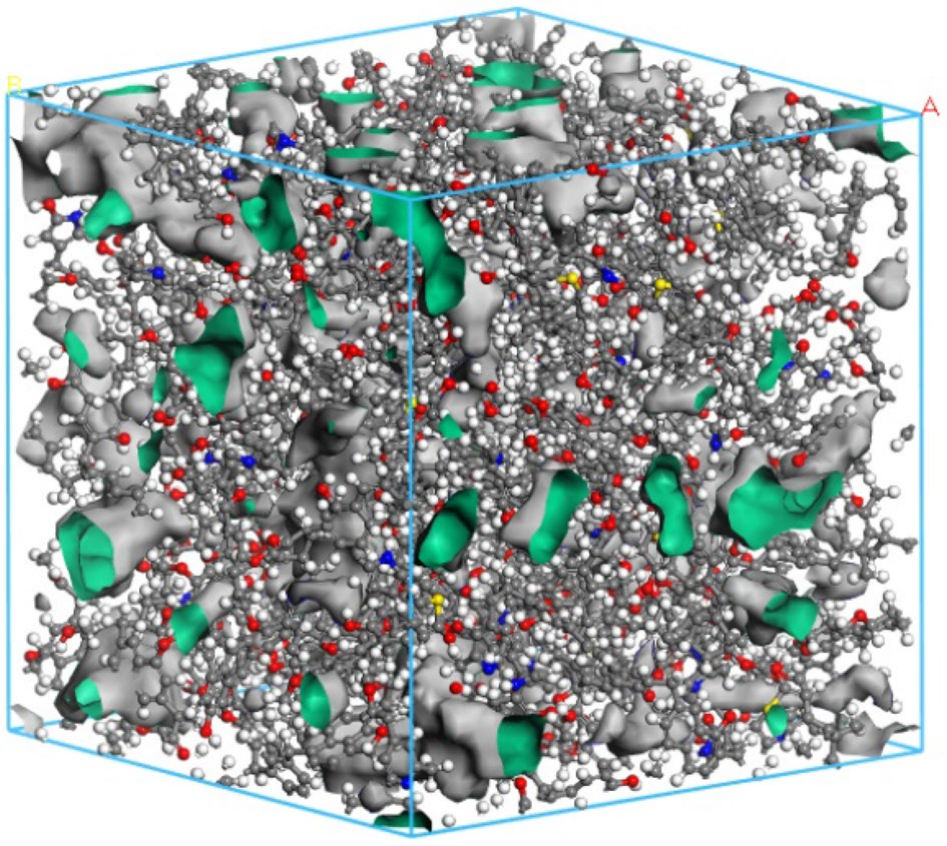
Macromolecular structure cell model of the QGP coal.

The Atom Volumes and Surfaces module in MS is adopted to complete the following settings: set the radius of Connolly probe as 0.13 nm (the molecular dynamics radius of He), select Connolly surface and Solvent surfaces, set the mesh resolution as 0.75 Å, the VDW scale factor as 1.0 Å and the Max. solventradiu as 2.0 Å. In this case, the size and molecular formula of macromolecular structure cell model of the QGP coal are determined to be A = B = C = 4.12442n and C_3075_H_2715_N_45_O_435_S_15_, respectively. The model is displayed in Fig. 11.

The cell model and pore size distribution of the coal sample show that the coal macromolecular structure is composed of two parts: the volume of the atomic skeleton and the free volume (i.e., the microporous pore structure in this paper). In the molecular structures of different coal samples, the micro pores feature non-homogeneous distribution and complex pore connectivity. To be specific, there are both relatively independent micropores and complex micropore networks composed of multiple pores.

## Conclusions

This paper takes the coal sample from QGP Coal Mine as the study object. Through a series of experiments and fitting analysis, the following conclusions were drawn:

Through proximate/ultimate analysis, it is concluded that the QGP coal sample belongs to long flame coal with a high content of oxygen and a low content of sulfur. Based on modern physicochemical characterization methods, including XPS, FTIR and ^13^C-NMR, the following data are collected: the aromaticity of the coal sample is 73.64%; The ratio of bridge aromatic carbon to peripheral carbon is 0.198; The relative area scale of C–O stretching vibration in the phenol, alcohol, ether, phenoxy and ester of oxygen-containing functional groups is 37.22%; The relative area scale of carbonyl is 6.93%; The benzene rings are mainly disubstituted, accounting for 36.48%; The content of aliphatic hydrocarbons is mainly of symmetrical –CH_X_ stretching vibration, and the relative area scale is 37.33%. C_5_H_5_N and C_4_H_5_N are the main existing forms of nitrogen in the QGP coal sample, playing a dominant role, while the content of –N–(CH_3_)_3_ and N_x_O_y_ are relatively low; The sulfur exists in the QGP coal sample mainly in the form of thiophene, sulfone and sulfoxide, and less in the form of inorganic sulfur.

Based on the structural parameters of the QGP coal sample, the macromolecular model of the QGP coal sample was constructed with the aid of the chemical drawing software Kingraw and then imported into the MestReNova software. By constantly adjusting the positions and connection modes of various groups in the coal molecule, the aromatic unit and aromaticity were kept unchanged. Finally, the calculated ^13^C-NMR spectrum of the constructed model was compared with the experimental one and the molecular formula of the QGP coal sample was finally determined as C_205_H_181_O_29_N_3_S.

In the macromolecular structure model of the QGP coal sample optimized through molecular dynamics, annealing dynamics and quantum chemistry, the arrangement of aromatic layers tends to be ordered with an increasing layer spacing, showing a strong three-dimensional effect. The calculated ^13^C-NMR spectrum is consistent with the experimental one, which verifies the rationality of the constructed coal macromolecular model.

The MS software was used to base the constructed coal macromolecule model on the molecular probe method using the atomic volume and surface module in MS to complete the following settings: set the Connolly probe radius to 0.13 nm (molecular dynamics radius of He), select the Connolly surface and solvent surface, set the grid resolution to 0.75 Å, VDW scale factor to 1.0 Å, and Max. solventradiu was 2.0 Å, and the microporous structure characteristics of the coal macromolecule model were obtained.

## Data Availability

All data generated or analysed during this study are included in this published article (and its Supplementary Information files).
